# *Toxoplasma gondii* infection-induced host cellular DNA damage is strain-dependent and leads to the activation of the ATM-dependent homologous recombination pathway

**DOI:** 10.3389/fcimb.2024.1374659

**Published:** 2024-03-08

**Authors:** Lisbeth Rojas-Barón, Carlos Hermosilla, Anja Taubert, Zahady D. Velásquez

**Affiliations:** Institute of Parasitology, Biomedical Research Center Seltersberg, Justus Liebig University Giessen, Giessen, Germany

**Keywords:** *Toxoplasma gondii*, DNA damage, chromosome instability, micronuclei, double-stranded DNA breaks, DNA repair pathways

## Abstract

*Toxoplasma gondii* is a globally occurring apicomplexan parasite that infects humans and animals. Globally, different typical and atypical haplotypes of *T. gondii* induce varying pathologies in hosts. As an obligate intracellular protozoon, *T. gondii* was shown to interfere with host cell cycle progression, leading to mitotic spindle alteration, chromosome segregation errors and cytokinesis failure which all may reflect chromosomal instability. Referring to strain-dependent virulence, we here studied the potential of different *T. gondii* strains (RH, Me49 and NED) to drive DNA damage in primary endothelial host cells. Utilizing microscopic analyses, comet assays and γ-H2AX quantification, we demonstrated a strain-dependent induction of binucleated host cells, DNA damage and DNA double strand breaks, respectively, in *T. gondii*-infected cells with the RH strain driving the most prominent effects. Interestingly, only the NED strain significantly triggered micronuclei formation in *T. gondii*-infected cells. Focusing on the RH strain, we furthermore demonstrated that *T. gondii*-infected primary host cells showed a DNA damage response by activating the ATM-dependent homologous recombination (HR) pathway. In contrast, key molecules of the nonhomologous DNA end joining (NHEJ) pathway were either not affected or downregulated in RH-infected host cells, suggesting that this pathway is not activated by infection. In conclusion, current finding suggests that *T. gondii* infection affects the host cell genome integrity in a strain-dependent manner by causing DNA damage and chromosomal instability.

## Introduction

*Toxoplasma gondii*, the causative agent of toxoplasmosis, is an obligate apicomplexan parasite capable of infecting all nucleated cells of all warm-blooded animals (Tenter et al., 2000; Brunet et al., 2008; Dubey, 2009). In humans, this parasite infects up to a third of the global population, but infection in most cases remains asymptomatic resulting in chronicity (Bigna et al., 2020; Fernández-Escobar et al., 2022). However, pregnant and immunocompromised individuals represent risk groups and may develop abortion and severe neurological/ocular disorders, respectively (Jones et al., 2003; Shobab et al., 2013; Robert-Gangneux et al., 2018; Rostami et al., 2020).

As an obligate intracellular parasite, *T. gondii* has developed multiple mechanisms to manipulate a broad range of biological processes in its host cell, such as metabolism, autophagy or apoptosis, to guarantee its intracellular replication and survival (Brunet et al., 2008; Zhuang et al., 2020). Also, it has been described that *T. gondii*-driven cell cycle perturbation thereby mainly documenting an arrest in early cell cycle phases, i. e. before progression to mitosis. Human foreskin fibroblasts infected with *T. gondii* tachyzoites accumulate at the G2/M boundary (Molestina et al., 2008) while human trophoblast cell line and in human dermal fibroblasts (Brunet et al., 2008) or even both (L6 rat myoblast cell line (Kim et al., 2016), were reported to be arrested in the G2-phase. Immortalized human fibroblasts, HFF, were arrested in the S-phase after 6 h p.i. with *T. gondii* tachyzoites (Pierre-Louis et al., 2022). The G2 phase arrest in human dermal fibroblasts or a human trophoblast cell line was linked to the downregulation of cyclin B1 (Brunet et al., 2008). Interestingly, cyclin B1 gene promotor silencing was shown to be mediated by *T. gondii*-secreted ROP16 targeting host cellular transcription factor UHRF1 and triggering host cell cycle arrest by inducing a series of epigenetic reactions (deacetylation, methylation of histone H3 around the cyclin B1 promotor) (Sabou et al., 2019). Cell cycle arrest during S phase at G2/M boundary in *T. gondii*-infected human foreskin fibroblasts was accompanied by a delayed or lacking increase of cyclin A and cyclin B1 in combination with an early elevation of cyclin E1 when compared to EGF stimulation, thereby indicating a missing exit from S phase and failure to progress toward mitosis in *T. gondii* infection (Molestina et al., 2008). This kind of cell cycle perturbation depended on parasite viability and proliferation since replication-defective tachyzoites hardly influenced the host cell cycle (Molestina et al., 2008). In the murine RAW264.7 cell line, *T. gondii* infections caused an enhanced proportion of polyploid cells (8n), most probably reflecting DNA replication without subsequent cytokinesis (Franco et al., 2016). Nonetheless *T. gondii* not only modulates the host cell cycle but also host cell chromosome segregation and cytokinesis in infected primary endothelial cells (Velásquez et al., 2019). In general, chromosome segregation and cytokinesis impairment are reported to be related to chromosomal instability (Holland and Cleveland, 2009), a phenomenon which is linked to a variety of chromosomal aberrations, such as loss, gain or disarrangement of chromosomes (Bakhoum and Cantley, 2018). Likewise, chromosomal instability is known to induce DNA damage, resulting in DNA strand breaks and cellular stress during DNA replication (Wilhelm et al., 2020). However, to date, little is known on *T. gondii*-driven DNA damage in host cells and even less data is available on mechanisms, which are activated by host cells in response. Recent data showed that *T. gondii* RH strain-infected tumor cells experienced parasite-driven DNA double-strand breaks and reacted by activating the homologous recombination repair pathway (Zhuang et al., 2020). Given that DNA damage of host cells may be involved in *T. gondii* pathogenicity, strain-dependent reactions seem of high interest, but related data are currently missing. It is well-known that *T. gondii* strains show significant genetic, phenotypic and pathogenic diversity. Nowadays, three main lineages (types I-III) are described according to their virulence and mortality for laboratory mice (Fernández-Escobar et al., 2021). *T. gondii* strains circulating in Europe mainly belong to type II strains and, to a less extent, to type III strain, both in humans and animals (Khan et al., 2007; Lorenzi et al., 2016; Fernández-Escobar et al., 2022). The *T. gondii* RH strain, widely used in *Toxoplasma*-related research, belongs to type I strains, which are classified as highly virulent leading to widespread parasite dissemination and lethal infection in mice (100% cumulative mortality) (Sanchez and Besteiro, n.d.). To address strain-dependent host cell DNA damage and chromosomal instability induction, we here tested representatives of haplotype I-III by using RH (type I), Me49 (type II) and NED (type III) strains.

To avoid artifacts eventually driven by the immortalization status of (tumor) cells, we here studied reactions of primary (endothelial) host cells. Overall, strain-related data partially mirrored strain pathogenicity. Thus, RH strain infections most potently induced the formation of both, binucleated host cells and host cellular DNA damage foci whilst the Me49 strain failed to do so. Interestingly, NED infections also moderately triggered binucleated cells, DNA damage foci and micronuclei formation. Focusing on DNA damage-related responses in RH strain infections, especially the ATM-related branch of the homologous recombination (HR) pathway was activated in primary host cells whilst molecules of the non-homologous end joining (NHEJ) pathway were either not affected or even found downregulated.

## Materials and methods

### Primary bovine umbilical vein endothelial cell isolation and culture

Primary bovine umbilical vein endothelial cells (BUVEC) were isolated from umbilical veins obtained from calves born by *sectio caesarea* at the Justus Liebig University Giessen, Germany. Umbilical cords were maintained at 4°C in 0.9% HBSS-HEPES buffer (pH 7.4; Gibco, Grand Island, NY, USA) supplemented with 1% penicillin (500 U/mL; Sigma St. Louis, MO, USA) and streptomycin (500 µg/mL; Sigma) for a maximum of 12 h before use. Isolation of endothelial cells was performed by using 0.025% collagenase type II (Worthington Biochemical Corporation) suspended in Pucks solution (Gibco) and infused into the lumen of ligated umbilical veins for 20 min at 37°C in 5% CO_2_ atmosphere. After gently massaging umbilical veins, the cell suspension was collected in medium and supplemented with 1 mL fetal calf serum (FCS; Gibco) to inactivate collagenase. After two washes (350 *xg*, 12 min, RT), cells were resuspended in complete endothelial cell growth medium (ECGM, PromoCell, supplemented with 10% FCS), plated in 25 cm^2^ tissue plastic culture flasks (Greiner) and incubated at 37°C and 5% CO_2_ atmosphere. BUVEC were cultured in modified ECGM medium [ECGM, diluted at 30% in M199 medium, supplemented with 5% FCS (Greiner) and 1% penicillin and streptomycin] with medium changes every 2-3 days. All biological isolates were used for *in vitro* experiments at a maximum of 4 passages. Experiments on bovine primary endothelial cells and parasites were performed following the permission of the Institute of Parasitology to work with biological agents up to risk class 3** [allowance according to §16 BiostoffVO, Az. GI 000056837, approved by the regional commission of Giessen (Regierungspräsidium Gieβen)], Institutional Ethics Commission of Justus Liebig University Giessen (Germany), and under the current European Animal Welfare Legislation: ART13TFEU.

### Parasite maintenance

Tachyzoites of *Toxoplasma gondii* (RH, Me49, NED strains corresponding to haplotypes I, II, III, respectively), were maintained by serial passages in HFF cells (human foreskin fibroblast), using DMEM (1X) + GlutaMAX medium (Gibco 61965-026) supplemented with 10% FCS, 1% penicillin (500 U/mL; Sigma St. Louis, MO; USA) and streptomycin (500 µg/mL; Sigma). Tachyzoite stages were obtained by scraping the monolayer and filtering through a syringe with a 25G needle to release tachyzoites from their parasitophorous vacuoles in the host cells. The parasite suspension was centrifuged at 400 *xg* for 15 seconds to remove cell debris and the supernatant containing tachyzoites was collected. A second centrifugation step was performed to sediment the parasites at 400 *xg* for 10 min. Tachyzoites were counted in a Neubauer chamber, suspended in a modified ECGM medium, and used for BUVEC infections and subsequent experiments.

### Comet assays

For cellular DNA damage assessment, three BUVEC isolates were infected with *T. gondii* RH, NED or Me49 tachyzoites using an MOI of 1:2. At 24 h p. i., non-infected and infected cells were gently removed from plates by scraping and centrifuged at 700 *xg* for 2 min. The cell pellet was washed with 1X ice-cold PBS buffer (magnesium and calcium-free). Cells were resuspended at 1x10^5^ cells/mL in ice-cold PBS and analyzed via comet assays according to the manufacturer´s instructions (Abcam, ab238544). In brief, cells were first encapsulated in a low melting agarose suspension at a 1/10 ratio (v/v) and transferred onto the top of an agarose-based layer previously prepared in special glass comet slides, thereby maintaining the cell suspension at 37°C to avoid gelation. After gelation, cell samples were incubated in 1X lysis buffer [NaCl, EDTA solution, 10X lysis solution (provided by manufacturer), DMSO] for 1 h at 4°C to remove cell membranes, cytoplasm and nucleoplasm, and to solubilise nuclear packaging proteins. Thereafter, samples were treated with a pre-chilled alkaline solution (300 mM NaOH, 1 mM EDTA) for 30 min at 4°C, allowing DNA loops to be unwound. Samples were then subjected to electrophoresis at 12 V (1 volt/cm according to the chamber used), 240 mA for 30 min at 4°C under alkaline conditions, washed three times with pre-chilled destilled water for 2 min and once with cold 70% ethanol for 5 min. In a final step, DNA was visualized by 1X Vista Green DNA staining (15 min at RT), an intercalating DNA dye provided by the manufacturer. Based on their differential migratory behavior, intact DNA (= “comet head”) can be distinguished from DNA with single-stranded and double-stranded DNA breaks, resulting in “comet tail” structures. Comets (head + tails) were analyzed by the OpenComet Software allowing for automated analysis of comet assay images. The DNA damage was quantified by measuring the displacement between intact nuclear DNA (comet head) and the resulting tail, resulting from single- and double-strand DNA breaks. Hence, the tail length (µm) was graphed for this assay.

### Immunofluorescence assays

Three BUVEC isolates were seeded in 12-well plates with 12 mm coverslips previously precoated with fibronectin (1:400, Sigma: F1141). At subconfluency, BUVEC were infected with *T. gondii* RH, Me49 or NED tachyzoites at an MOI of 1:2. At 24 h p. i., samples were fixed in 4% paraformaldehyde (PFA, 15 min, RT) and washed three times in 1X PBS buffer. Samples were then blocked/permeabilized (1X PBS buffer, 3% BSA, 0.3% Triton X-100, all from Carl Roth) for 1 h at RT and incubated in primary antibody solutions (**Table 1**) at 4°C in a humidified chamber, overnight. Thereafter, samples were washed three times with 1X PBS and incubated in secondary antibody solutions (**Table 1**) for 30 min at RT and darkness. Cell nuclei were stained with 4´, 6-diamidin-2-phenylindole (DAPI) present in mounting medium solution (Fluoromount G-DAPI, Thermo Fischer Scientific, Cat. N° 495952) and analyzed with a ReScan Confocal microscope instrumentation (RCM 1.1 Visible, Confocal.nl) combined with a Nikon Ti-2 Eclipse microscope.

**Table 1:** Primary and secondary antibodies used for immunofluorescencs assays.

Antigen	Company	Cat. number	Origin/reactivity	Dilution
Primary antibodies				
*T. gondii*	ThermoFisher	PA1-7256	Goat	1:100
H2AvD (pS137)	Cell Signalling	80312	Mouse	1:300
H2AvD (pS137)	Rockland	600-401-914S	Rabbit	1:300
	Secondary antibodies			
Antigen/Conjugate	Company	Cat. number	Host/target	Dilution
Alexa Fluor 488	ThermoFisher	A11055	goat	1:500
Alexa Fluor 594	ThermoFisher	A-11058	goat	1:500
Alexa Fluor 594	ThermoFisher	R37117	rabbit	1:500
Alexa Fluor 647	ThermoFisher	A-21244	rabbit	1:500
Alexa Fluor 647	ThermoFisher	A-21235	mouse	1:500

### Protein extraction from *T. gondii*-infected host cells

Six primary BUVEC isolates were infected with *T. gondii* RH tachyzoites (MOI 1:2). At 24 h p. i., cells were washed with 1X PBS buffer, detached from the plate using trypsin/EDTA solution [0.25% (w/v) trypsin; 0.53 mM EDTA, 37°C, 5 min] and pelleted at 400 *xg* for 5 min. The cell pellet was washed with 1X PBS buffer and resuspended in RIPA buffer (50 mM Tris-HCl, pH 7.4; 1% NP-40; 0.5% Na-deoxycholate; 0.1% SDS; 150 mM NaCl; 2 mM EDTA; 50 mM NaF; all Roth) supplemented with a protease inhibitor cocktail (Sigma-Aldrich), 1 mM sodium orthovanadate tyrosine phosphatase inhibitor (Abcam ab120386) and 1 mM phenylmethylsulphonyl fluoride, a serine protease inhibitor (Abcam ab141032). Protein extracts were sonicated for five cycles of 20 s sonication and 20 s resting and then centrifuged (10,000 *x g*, 10 min, 4°C) to sediment intact cells, membranes, and nuclei. The supernatants were analyzed for protein content via BCA Protein Assay (Pierce BCA Protein Assay Kit, Thermo Scientific, cat. number 23225) following the manufacturer’s instructions. Protein concentration was quantified in a Varioskan plate reader by measuring the absorbance at 562 nm.

### SDS-PAGE and immunoblotting

Protein extracts were diluted in loading buffer with 6 M urea (10% SDS, 12.5% 2-mercaptoethanol, 25% glycerol, 150 mM Tris-HCl pH 6.8) and boiled at 95°C for 5 min. Samples (40 µg protein/slot) were loaded on 6% polyacrylamide gels and subjected to SDS-PAGE electrophoresis (200 V; approx. 50 min, BioRad). Proteins were transferred to PVDF membranes (Millipore) at 300 mA for 2 h in a wet-tank transfer system (BioRad) and then blocked for 1 h at RT [3% BSA in TBS buffer (50 mM Tris-HCl, 150 mM NaCl; pH 7.6)]. Afterwards, membrane-bound proteins were reacted with primary antibodies (diluted in blocking solution: TBS buffer, 0.1% Tween-20, 3% BSA, 4°C, overnight) directed against key proteins of the homologous recombination repair pathway (ATM, BRCA1, p-BRCA1, BRCA2, Rad54, Mre11, p-Mre11, p95NBS1), non-homologous end joining (DNA-PKcs, p-DNA-PKcs, Ku70, Artemis, DNA ligase IV) and vinculin (used as a loading control for the normalization of the samples) (**Table 2**). Thereafter, the membranes were washed three times with TBS-Tween (0.1%) and incubated in secondary antibody solutions (**Table 2**) for 30 min at RT. After three washings in TBS-Tween (0.1%), protein detection was performed using an enhanced chemiluminescence detection system (ECL Prime, Amersham). Images were taken using the Science Imaging Instrument (INTAS) applying the INTAS ChemoStar Imager software. A protein ladder was used to estimate protein masses (HiMark Pre-stained Protein Standard #LC5699, Thermo Fisher Scientific). Protein band intensities were analyzed by the Fiji Gel Analyzer plugin.

**Table 2:** Primary and secondary antibodies used for Western Blotting.

Antigen	Company	Cat. number	Origin/reactivity	Dilution
Primary antibodies				
ATM (D2E2)	Cell Signalling	2873	Rabbit	1:1000
BRCA1	Cell Signalling	14823	Rabbit	1:1000
p-BRCA1	Cell Signalling	9009	Rabbit	1:1000
BRCA2	Cell Signalling	10741	Rabbit	1:1000
Rad54	Cell Signalling	15016	Rabbit	1:1000
Mre1l	Cell Signalling	4887	Rabbit	1:1000
p-Mre1l	Cell Signalling	4859	Rabbit	1:1000
P95/NBS1	Cell Signalling	14956	Rabbit	1:1000
DNA-PKcs	Cell Signalling	38168	Rabbit	1:1000
Ku70	Cell Signalling	4588	Rabbit	1:1000
Artemis (D708V)	Cell Signalling	13381	Rabbit	1:1000
DNA ligase IV	Cell Signalling	14649	Rabbit	1:1000
Vinculin	Santa Cruz	sc-73614	Mouse	1:500
	Secondary antibodies			
Antigen/Conjugate	Company	Cat. number	Host/target	Dilution
Goat anti-mouse IgG Peroxidase conjugated	Pierce	31430	Goat/mouse	1:40,000
Goat anti-rabbit IgG Peroxidase conjugated	Pierce	31460	Goat/rabbit	1:40,000

### Quantification of binucleated and γH2AX-positive cells

All quantifications were performed in three biological BUVEC replicates infected with tachyzoites of *T. gondii* RH, Me49 or NED strains. Samples were collected at 24 h p. i. and stained for DNA (nuclear staining, DAPI, blue, Invitrogen), DNA damage foci (γH2AX, green, **Table 1**) and tachyzoites (anti-*Toxoplasma* antibodies, red, **Table 1**). For immunofluorescence assays, please refer to the section above. In all experimental settings, five random images were acquired at 60X with an epifluorescence microscope (Nikon Ti-2 Eclipse) and a total of 1800 cells were analyzed per condition. For binucleated cell estimation, the total number of binucleated cells was normalized by the total number of cells in the same field of view. The DNA damage quantification was performed by the detection of γH2AX protein inside of the nuclear area. The histone variant H2AX is phosphorylated in Ser 139 when cells display DNA double strand breaks induction forming DNA damage foci inside the nucleus that can vary in number from one to hundred. Therefore, we consider a positive cell to be one that shows at least one focus of DNA damage positive for γH2AX. First, we proceeded with the segmentation of the DAPI channel in each image and the respective ROIs were merged with the green channel (γH2AX staining) to allocate the nuclear region in the image with the DNA damage foci staining. Each γH2AX-positive cell was counted as one independently on the number of DNA damage foci per nucleus. All γH2AX-positive cells were counted and normalized to the total number of cells in the same field of view. Micronuclei were defined as roundish DAPI-positive structures localized in juxtaposition but separate from the nuclear area. The total number of micronuclei was counted and normalized to the total number of cells in the same field of view.

### Image acquisition and reconstruction

Fluorescence images were acquired with a ReScan Confocal microscope instrumentation (RCM 1.1 Visible, Confocal.nl) equipped with a fixed 50 µm pinhole size and combined with a Nikon Ti-2 Eclipse microscope equipped with a motorized Z-stage (DI1500, Nikon). The RCM unit was connected to a Toptica CLE laser with the following excitations: 405/488/561/640 nm. Images were taken via an sCMOS camera (PCO edge) using a CFI Plan Apochromat X60 lambda-immersion oil objective (NA 1.4/0.13; Nikon). The instrument was operated by the NIS-Elements software (version 5.11). To calculate the total number of cells and the number of binucleated cells present within one cell layer, all images were first segmented using the Otsu thresholding algorithm. Identical brightness and contrast conditions were applied for each data set within one experiment. The total number of cells was obtained using the Fiji plugin “Analyze particles” applying a size of 10 µm.

### Statistical analysis

The data were expressed as mean ± SD from independent experiments. For cell number quantification experiments, one-way analysis of variance (non-parametric ANOVA) with Kruskal-Wallis´s post-test was performed using GraphPad Prism 9.3.1 software applying a significance level of 5%. For WB-based analyses, unpaired two-tailed *t*-tests were performed comparing controls vs infected cells at a 95% confidence interval. All graphs and statistical analyses were performed using GraphPad Prism 9 software.

## Results

### *T. gondii*-driven host cell DNA damage and binucleated cell formation is haplotype-dependent

**Figure 1:**
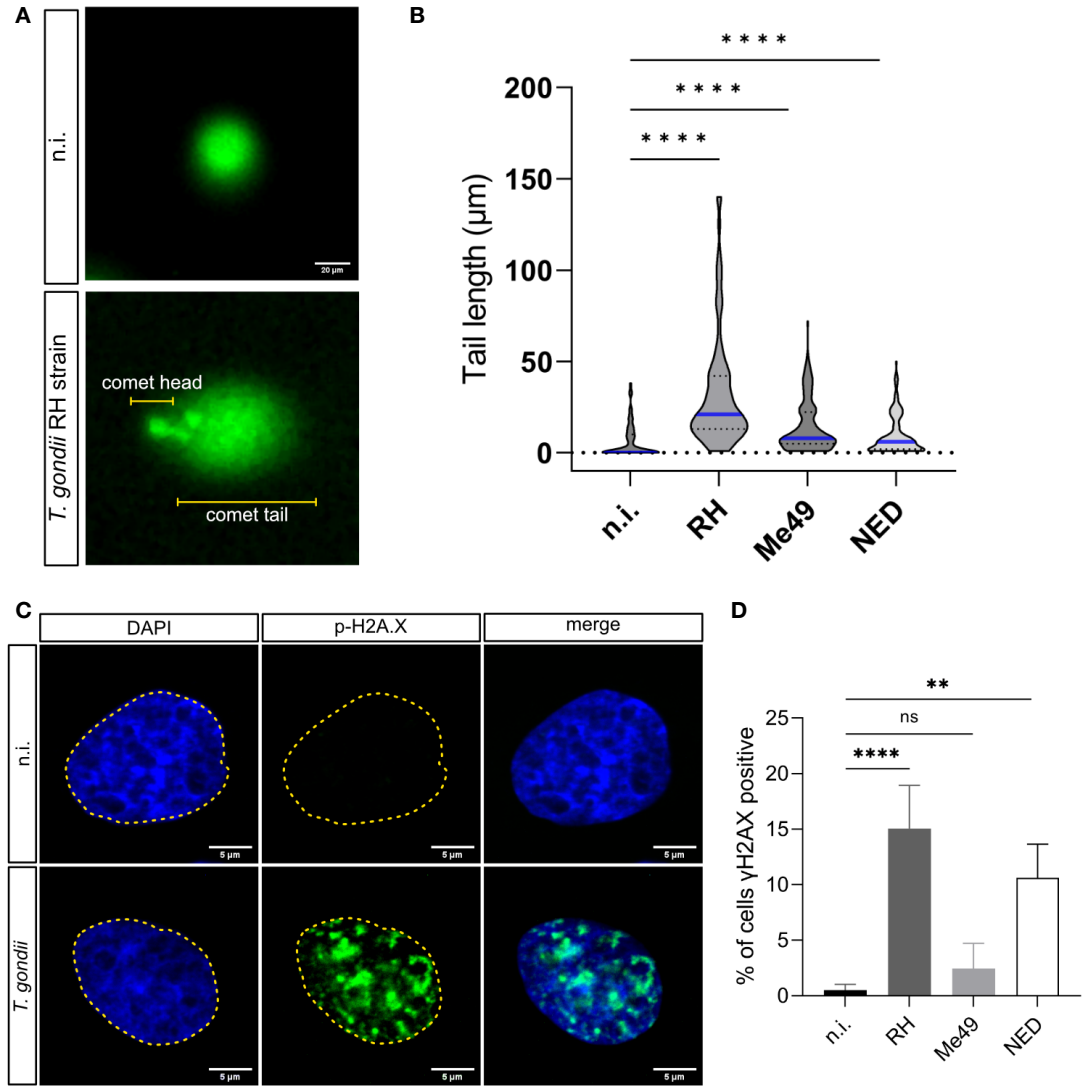
Induction of DNA damage by *Toxoplasma gondii* RH, Me49 and NED infections in primary bovine endothelial cells. BUVEC monolayers (*n* = 3) were infected with RH, Me49 or NED tachyzoites (MOI 1:2; 24 h p.i.) to analyze infection-driven DNA damage using comet assays. **(A)** Exemplary illustration of comet assays on *T. gondii* RH-infected and uninfected cells. The round shape (comet head) identifies cells lacking DNA strand breaks while the comet tail reflects DNA with single or double DNA strand breaks. **(B)** The comet tail length was measured and plotted to compare *T. gondii* RH, Me49 and NED-infected cells with non-infected controls. The data showed that all *T. gondii* strains increased the number of host cells experiencing single- or double-stranded DNA breaks. **(C, D)** Detection of double-strand break-based DNA damage foci by γH2AX immunostaining. The data revealed a strain-dependent induction of DNA damage foci. The scale bar represents 20 or 5 μm. ns: not significant, **p < 0.01, ****p < 0.0001.

*T. gondii* RH infections have been reported to induce the host cell cycle arrest and to cause alterations in chromosome segregation and cytokinesis in host cells (Velásquez et al., 2019). These aberrant processes include chromosomal instability, resulting from chromosome loss, gain or inadequate DNA rearrangements (Bakhoum and Cantley, 2018). Chromosomal instability can trigger DNA damage by causing DNA strand breaks or by inducing cell stress during DNA replication (Wilhelm et al., 2020). Given that *T. gondii* indeed affects chromosomal segregation (Velásquez et al., 2019), we aimed here to analyze whether *T. gondii* infections induce host cellular DNA damage. In a first experimental approach, we analyzed the DNA strand integrity by using conventional comet assays. This method is well-accepted for general DNA damage detection in eukaryotic cells but cannot distinguish between single and double-strand breaks (Ostling and Johanson, 1984). Here, cells with intact DNA only show a comet head and - due to altered migratory behavior in the electric field - damaged DNA strands form the comet tail (**Figure 1A**). Current data revealed that *T. gondii* RH, NED and Me49 infections of BUVEC in principle all resulted in a significantly increased proportion of cells experiencing DNA damage in comparison to non-infected controls (infected cells vs. controls for RH, NED and Me49: *p* ≤ 0.0001; p ≤ 0.0001; p≤ 0.0001, respectively, **Figure 1B**). However, by far, the strongest reactions were induced by the RH strain (**Figure 1B**).

Given that cellular damage responses induced by DNA single- or double-strand breaks involve different signaling pathways and protein factors (Ma and Dai, 2018) and considering that comet assays cannot distinguish between single- and double-strand breaks, we here applied a second experimental approach by assessing γ-H2AX signals in *T. gondii*-infected cells. In higher eukaryotic cells, DNA double-strand breaks trigger the phosphorylation of the histone H2A variant H2AX at serine 139 to generate γ-H2AX (Kinner et al., 2008). Nuclear γ-H2AX signals are therefore used as a marker for double-strand break events being represented as DNA damage foci. In the current study, BUVEC were infected with RH, Me49 or NED strains of *T. gondii*, stained for DNA by DAPI to identify the nuclear area and co-labeled for γ-H2AX at 24 h p. i. (**Figure 1C**). Overall, the proportion of cells experiencing DNA damage foci (= γ-H2AX-positive BUVEC) increased with parasite infections, a finding that revealed *T. gondii* haplotype-dependent. Thus, a significant parasite-driven induction of DNA double-strand breaks was detected in both RH- (15.1% positive cells, *p <*0.0001) and NED- (10.6% positive cells, *p* = 0.0001) infected cells. In contrast, only 2.4% of Me49-infected cells showed DNA damage foci thereby failing to differ significantly from non-infected controls (0.5%, **Figure 1D**). Given that the - by far - strongest effects were again found in RH strain-infected cells, this finding may mirror strain pathogenicity (**Figure 1D**). Altogether, these findings suggest that infections with the *T. gondii* strains NED and RH both induced double strand DNA breaks while Me49 infections do not.

**Figure 2:**
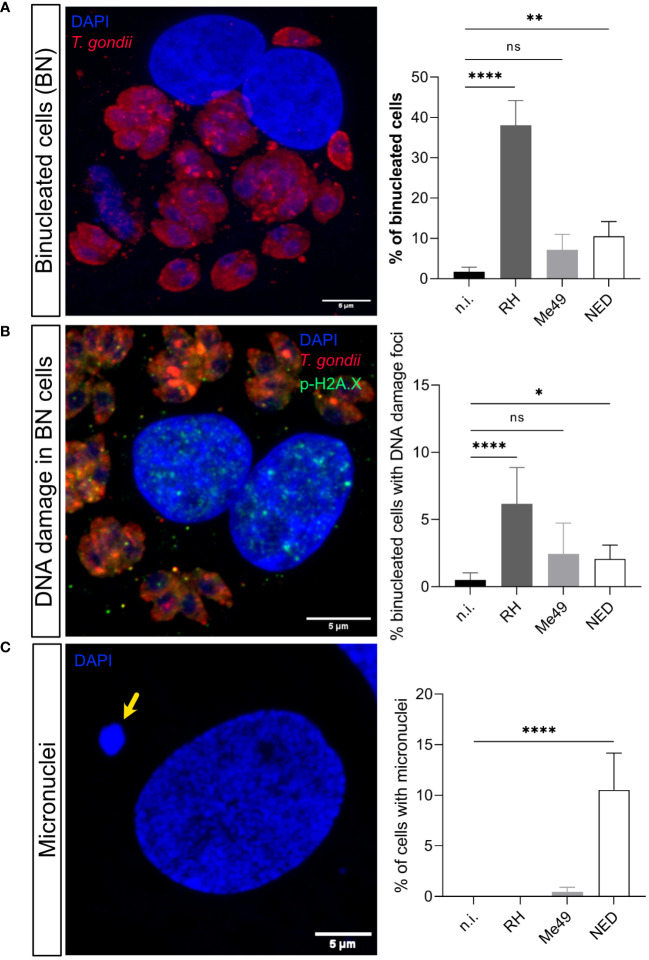
Effects of *Toxoplasma gondii* RH, Me49 and NED infections on BUVEC in binucleated host cell and micronuclei formation. BUVEC isolates (*n* =3) were infected with RH, Me49 or NED tachyzoites at an MOI 1:2 during 24 h **(A)** The presence of binucleated cells was estimated in *T. gondii-*infected cells. Data showed that RH and NED strains induced host binucleated phenotypes at different quantitative levels whilst this phenomenon was not observed in Me49-infected cells or controls. **(B)** The percentage of DNA damage foci-positive binucleated cells was analyzed showing that only RH and NED infections induced both findings at a time. **(C)** Detection of micronuclei formation indicated that exclusively NED-infected host cells revealed a significant increase. The scale bar represents 5 μm. ns: not significant, *p ≤ 0.05, **p < 0.01, ****p < 0.0001.

Chromosomal instability is structural or numerical due to chromosome segregation impairment, chromosome condensation defects or abnormal cytokinesis (Siri et al., 2021). Given that we demonstrated that *T. gondii-*induced DNA damage is haplotype-dependent, we also assessed the influence of these haplotypes on cytokinesis failure on the level of infection-driven binucleated host cell formation. In agreement to DNA damage foci-related data, only infections with the RH (38.1%, *p* < 0.0001) and NED (10.5%, *p* = 0.002) strains led to a significant increase in the formation of binucleated cells and, again, RH-infected cells showed the overall strongest effects (**Figure 2A**). Accordingly, when estimating the percentages of binucleated infected cells showing DNA damage foci (γ-H2AX-positive cells), a significantly increased proportion of 6.8% (*p <*0.0001) and 2.3% (*p* = 0.029) was found in RH- and NED-strain-infected BUVEC, respectively, whilst neither Me49-infected cells nor non-infected control binucleated cells showed a significant induction of DNA double-strand breaks (**Figure 2B**). Hence, these findings suggest that DNA damage may indeed be associated with the binucleated phenotype induced by *T. gondii*.

Besides causing cytokinesis impairment, chromosome condensation and segregation failures may also result in a lack or displacement of chromosomes or in the formation of chromosome bridges and micronuclei (Fenech et al., 2011; Ye et al., 2019; Siri et al., 2021). Micronuclei are typically represented by DAPI-positive globular structures which are localized close but separate from the cell nucleus. Here, we estimated the presence of micronuclei in RH-, Me49- and NED-infected BUVEC. As an interesting finding, a significantly enhanced proportion of host cells with micronuclei was exclusively found in NED-infected cells (10.5%, *p* < 0.0001) (**Figure 2C**).

### *T. gondii*-infected host cells activate the homologous recombination pathway (HR) to repair parasite-driven DNA damage

**Figure 3:**
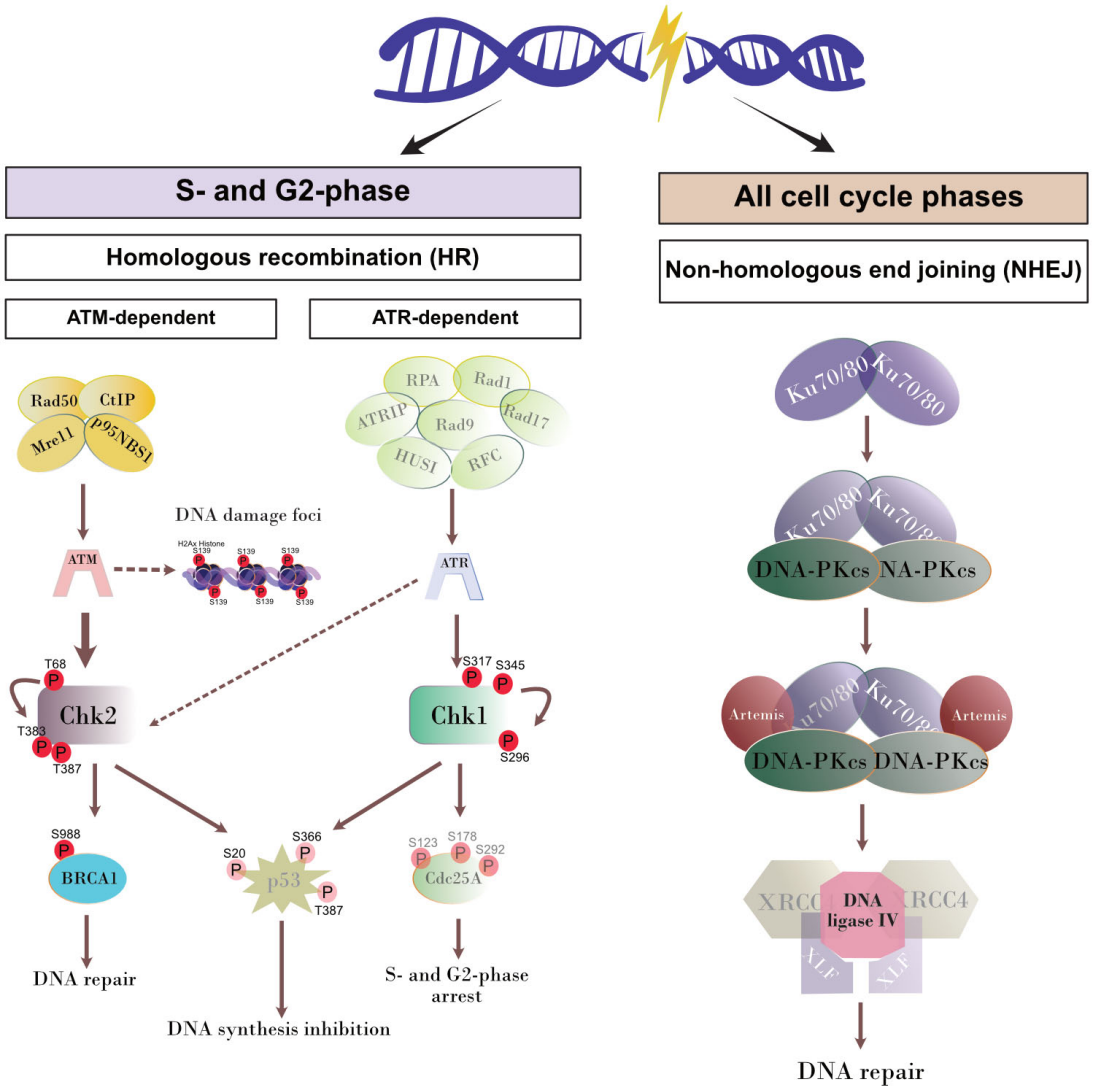
Schematic illustration of DNA repair pathways. The proteins of the pathways that were not analyzed in the current study are shown attenuated.

**Figure 4:**
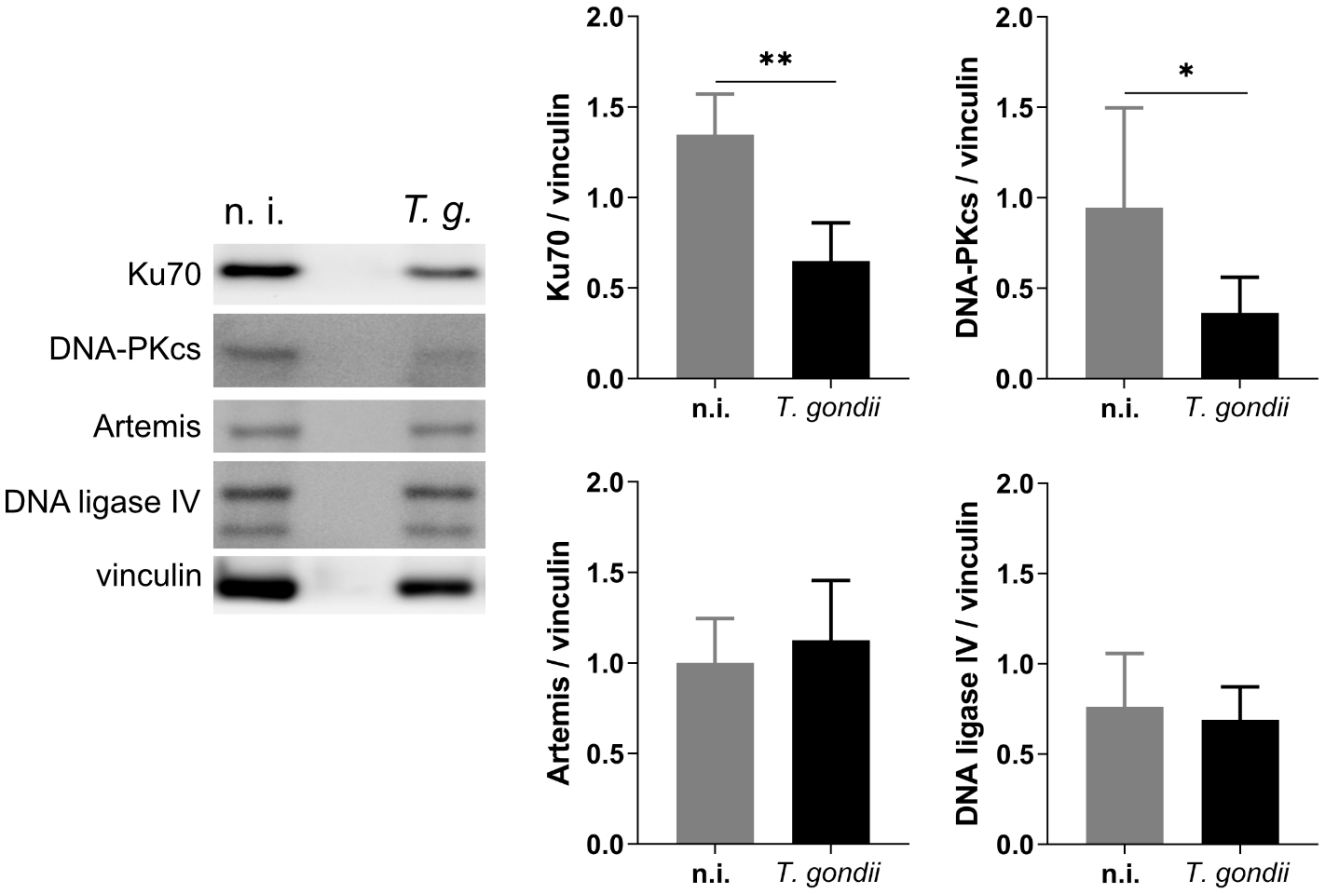
Effects of *T. gondii* RH infections on the NHEJ repair pathway. BUVEC isolates (*n* = 6) were infected with *T. gondii* RH strain (MOI 1:2). At 24 h p. i., samples were analyzed by Western Blotting for the expression of key proteins involved in the NHEJ pathway. The results showed that *T. gondii* RH infection does not activate the NHEJ pathway but downregulates the expression of Ku70 and DNA-PKcs. Bars represent the mean ± SD of six biological replicates. *p ≤ 0.05, **p < 0.01.

**Figure 5:**
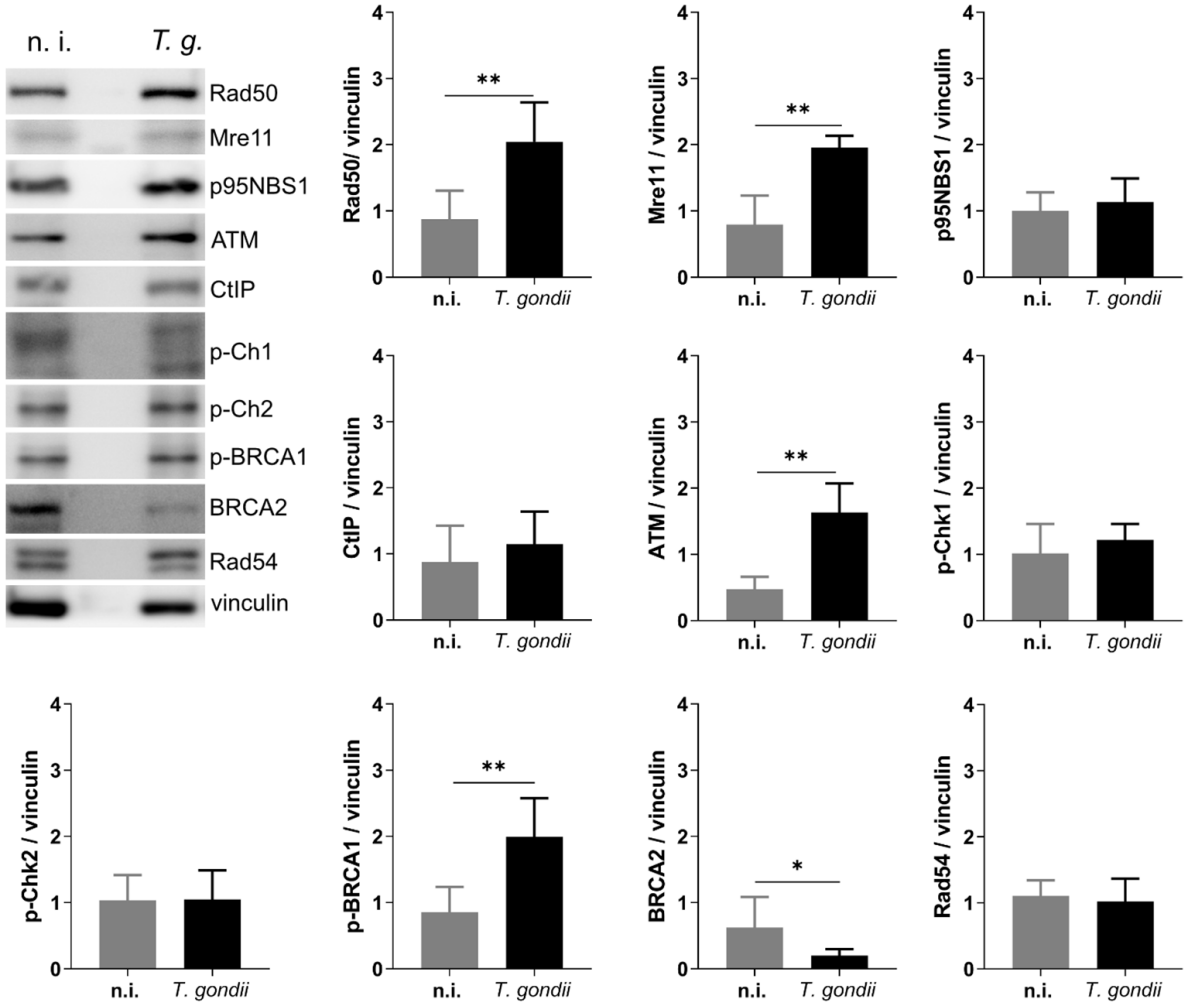
*T. gondii* infection activates the ATM-mediated HR repair pathway. BUVEC monolayers (*n* =6) were infected with *T. gondii* RH tachyzoites at an MOI 1:2. At 24 h p. i., protein extracts were analyzed via Western Blotting to analyze the expression of key proteins involved in the HR repair pathway. Results show an up-regulation of several proteins involved in the ATM-dependent HR pathway. Bars represent the mean ± SD of six biological replicates. *p ≤ 0.05, **p < 0.01.

Cells under genotoxic stress show a DNA damage response (DDR) and therefore activate different intracellular repair pathways depending on the type of DNA damage. Hence, DNA strand breaks either activate the homologous recombination (HR) or the non-homologous end joining (NHEJ) pathway. Interestingly, the HR pathway works cell cycle-dependent and primarily operates during the S- and G2-phase, whilst NHEJ mediated repair mechanisms are primarily activated after detection of DNA damage during the G1-phase. Nevertheless, this pathway may be activated throughout the cell cycle (Li and Heyer, 2008; Watanabe and Lieber, 2022) (**Figure 3**). Given that the current findings proved most prominent effects on DNA double-strand breaks in the case of the *T. gondii* RH strain, we here focused on RH strain infections and studied the expression of several key molecules of both, HR and NHEJ pathways by Western blotting (**Figure 4**). On a quantitative level we demonstrated that most molecules of the NHEJ pathway were not affected by infection (DNA ligase IV, artemis) or even down-regulated (Ku70, DNA-PKcs), indicating that *T. gondii* RH infections do not activate the NHEJ pathway (**Figure 4**). In contrast, several key molecules of the HR repair pathway were significantly induced in *T. gondii* RH-infected host cells (**Figure 5**). As schematically depicted in **Figure 3**, the HR repair pathway splits into two branches (ATM- and ATR-related pathways) depending on type of damaged DNA strand. Thus, the ATM-related pathway is mainly induced by DNA double-strand breaks, whilst the ATR-related pathway is activated by single strain breaks or by stalled replication forks. To distinguish the induction of ATM- or ATR-mediated signaling, we here studied the expression of several characteristic molecules of both pathways. Overall, *T. gondii* RH infections exclusively up-regulated molecules of the ATM-related pathways (ATM, Mre11, Rad50, and pBRCA) (**Figure 5**) whilst proteins involved in the ATR-related pathways either did not show any changes in expression (**Figure 5**) or – in case of BRCA2 – were even downregulated. Altogether, these findings confirm that *T. gondii* RH-infected host cells suffer from infection-driven DNA double-strand breaks and elicit a DNA damage response by activating the S-/G2-phase-related ATM-dependent branch of the HR pathway.

## Discussion

Genome integrity is an essential feature of cellular homeostasis and is a prerequisite for preserving the genetic information delivered to the offspring. Consequently, genome instability has a direct impact on cell, tissue or animal survival. Eukaryotic cells maintain genome integrity by activating different DNA damage response (DDR) pathways upon endogenous and environmental genotoxic stress promoting the repair of different types of DNA lesions. Moreover, activation of DDR controls cell cycle progression, thereby delivering time for cells to repair the damaged DNA (Campos and Clemente-Blanco, 2020; Petsalaki and Zachos, 2020). Previous results on primary host cells have shown that *T. gondii* infection affects the chromosomal stability of the host cells by inducing binucleated cell formation and chromosome segregation alterations (Velásquez et al., 2019). Interestingly, all of these features are hallmarks of chromosome instability (Geigl et al., 2008) and it appears likely that these events may be linked to the pathogenicity of *T. gondii* infections. On a global level, different *T. gondii* haplotypes with varying virulence occur. Whilst mainly the classical haplotypes I-III with an estimated moderate to low virulence circulate in Europe, atypical and mixed haplotypes eventually bearing high pathogenicity are spread in South America (Galal et al., 2022). To address a potential influence of different haplotypes on binucleated host cells, micronuclei formation and DNA-damage related cellular events, we here analyzed infection-driven effects of three different *T. gondii* strains (RH, Me49, NED strains corresponding to haplotypes I, II, III, respectively) on primary host endothelial cells.

In all experiments we worked with different donors of primary endothelial host cells to avoid cell cycle-related artifacts that may be present in permanent cell lines or in cells of tumor origin. Considering the three *T. gondii* strains we showed in a first experimental series that the RH strain - which is reported as the most pathogenic one among these three strains - most prominently induced the formation of binucleated cells thereby confirming recent RH-related findings and indicating cell cycle impairment (Velásquez et al., 2019). Interestingly, infections-driven binclucleated cell formation proved strain-dependent, since the NED strain triggered this event on a minor level whilst ME49 infections had no significant effect on binucleated phenotypes. Given that binucleated cell formation may be linked to chromosome instability and DNA damage, we then assessed a general overview on the presence of DNA damage in infected host cells via comet assays. Here, we confirmed strain-dependent effects and showed that the strongest DNA damage was again driven by the *T. gondii* RH infections. The NED strain induced low levels of DNA damage whilst the Me49 strain failed to do so. Given that comet assays cannot distinguish between single- and double-strand breaks, we additionally analyzed the presence of γ-H2AX-based DNA damage foci, which are indicative for DNA double-strand breaks. In agreement to the data mentioned above and referring to RH-, Me49- and NED-infected cell layers, strongest effects related to DNA damage foci were again attributed to the RH strain. The findings on the RH strain are in line to recent data of Zhuang et al (Zhuang et al., 2020), who reported on the induction of double-strand breaks in *T. gondii* RH-infected HeLa, HEK293T and Vero cells. Another hallmark of chromosome instability is the formation of so-called micronuclei. In contrast to our expectation, we here exclusively detected an enhanced proportion of host cells experiencing micronuclei formation in the case of NED strain infections.

Whenever a cell experiences genotoxic events and DNA damage, it activates a DNA damage response (DDR) via different repair pathways to preserve the correct genetic information to be passed to daughter cells. The repair pathway to be induced depends on type and extent of DNA insult, therefore single- and double-strand break repair pathways are both part of the DDR of a cell (**Figure 3**). Single-strand repair pathways include both excision repair modes (nucleotide excision repair and base excision repair) and mismatch repair. DNA double-strand insults may either induce the NHEJ or the homologous recombination (HR) repair pathways, the latter of which is linked to S- and G2 phases of cell cycle. Given that the current data confirmed DNA double-strand breaks in *T. gondii*-infected cells and revealed strongest effects in RH infections, we here studied the DNA damage response on the level of the HR and NHEJ pathways exclusively in RH-infected BUVEC. Overall, we found a *T. gondii*-induced activation of the HR pathway which indirectly supports former findings on a *T. gondii*-driven cell cycle arrest between S- and G2 phase in different types of host cells (Molestina et al., 2003; Brunet et al., 2008; Kim et al., 2016) and especially in BUVEC (Velásquez et al., 2019). In the case of the NHEJ pathway, the upstream molecules Ku70 and DNA-PKcs were found down-regulated in infected host cells indicating that this pathway may actively be blocked by parasite infection. Interestingly, Ku70 was reported to be involved in viral DNA recognition in human cells promoting type I and type III interferon/proinflammatory cytokine production (Sui et al., 2021), a reaction that is assumed to adverse affect *T. gondii* development. Moreover, Ku70 was shown to interact with Bax, thereby inhibiting Bax-mediated apoptosis (Cohen et al., 2004). Considering these aspects, infection-driven Ku70 downregulation might represent an DNA damage-independent event in *T. gondii*-infected host cells. Besides Ku70, the DNA-PKcs holoenzyme was also found downregulated in *T. gondii* RH infected cells. This protein forms a complex with Ku70/80 in response to DNA damage (Fell and Schild-Poulter, 2015). Moreover, it was shown that cells with low DNA-PKcs levels undergo accelerated cellular senescence (Liu et al., 2019). To our knowledge, there is currently no evidence that *T. gondii* modulates host cell senescence. The HR repair pathway includes different branches. In line with Zhuang et al. (2020) we here confirmed for a primary host cell type that ATM-mediated repair signaling is induced in *T. gondii* RH-infected BUVEC whilst the ATR-dependent pathway was not affected by infection.

Undoubtedly, the molecular mechanism of DNA damage induction driven by *T. gondii* seems of high interest, especially since it proved haplotype-dependent. However, the identification of parasite molecules triggering DNA double-strand breaks and their eventual molecular interactions with host cells are beyond the scope of the current study. Several *T. gondii*-secreted molecules, such as the ROP18 kinase, which was reported to interact with DNA repair-related host proteins (Cheng et al., 2012), may be good candidates in this respect and should be investigated in this respect in future.

Overall, the current findings underline the effects of *T. gondii* infections on host cell cycle and DNA integrity and demonstrate haplotype-dependent effects that may be linked to strain pathogenicity. Furthermore, current data confirm DNA damage responses of primary host cells on the level of ATM-dependent HR repair pathways.

## Data availability statement

The original contributions presented in the study are included in the article/supplementary material. Further inquiries can be directed to the corresponding author.

## Ethics statement

Experiments on bovine primary endothelial cells and parasites were performed following the permission of the Institute of Parasitology to work with biological agents up to risk class 3** [allowance according to §16 BiostoffVO, Az. GI 000056837, approved by the regional commission of Giessen (Regierungspräsidium Gieβen)], Institutional Ethics Commission of Justus Liebig University Giessen (Germany), and under the current European Animal Welfare Legislation: ART13TFEU. The study was conducted in accordance with the local legislation and institutional requirements.

## Author contributions

LR-B: Data curation, Formal analysis, Investigation, Methodology, Validation, Writing – review & editing. CH: Funding acquisition, Writing – review & editing. AT: Funding acquisition, Resources, Supervision, Writing – review & editing. ZV: Conceptualization, Data curation, Formal analysis, Investigation, Methodology, Software, Supervision, Validation, Writing – original draft, Writing – review & editing.
